# Advancements and Applications of Preimplantation Genetic Testing in In Vitro Fertilization: A Comprehensive Review

**DOI:** 10.7759/cureus.57357

**Published:** 2024-03-31

**Authors:** Sravya Gudapati, Kamlesh Chaudhari, Deepti Shrivastava, Seema Yelne

**Affiliations:** 1 Obstetrics and Gynaecology, Jawaharlal Nehru Medical College, Datta Meghe Institute of Higher Education and Research, Wardha, IND; 2 Nursing, Shalinitai Meghe College of Nursing, Datta Meghe Institute of Higher Education and Research, Wardha, IND

**Keywords:** comparative genomic hybridization (cgh), next-generation sequencing (ngs), assisted reproductive technology (art), genetic screening, in vitro fertilization (ivf), preimplantation genetic testing (pgt)

## Abstract

Preimplantation genetic testing (PGT) has become an integral component of assisted reproductive technology (ART), offering couples the opportunity to screen embryos for genetic abnormalities before implantation during in vitro fertilization (IVF). This comprehensive review explores the advancements and applications of PGT in IVF, covering its various types, technological developments, clinical applications, efficacy, challenges, regulatory aspects, and future directions. The evolution of PGT techniques, including next-generation sequencing (NGS) and comparative genomic hybridization (CGH), has significantly enhanced the accuracy and reliability of genetic testing in embryos. PGT holds profound implications for the future of ART by improving IVF success rates, reducing the incidence of genetic disorders, and mitigating the emotional and financial burdens associated with failed pregnancies and genetic diseases. Recommendations for clinicians, researchers, and policymakers include staying updated on the latest PGT techniques and guidelines, exploring innovative technologies, establishing clear regulatory frameworks, and fostering collaboration to maximize the potential benefits of PGT in assisted reproduction. Overall, this review provides valuable insights into the current state of PGT and its implications for the field of reproductive medicine.

## Introduction and background

Preimplantation genetic testing (PGT) refers to the screening of embryos conceived through in vitro fertilization (IVF) for genetic abnormalities before their transfer into the uterus [1]. The primary purpose of PGT is to identify genetic defects or chromosomal abnormalities in embryos, allowing for the selection of embryos with the highest likelihood of resulting in a successful pregnancy and healthy offspring. By analyzing the genetic material of embryos at the preimplantation stage, PGT aims to improve the chances of successful pregnancy and reduce the risk of genetic diseases or miscarriages [1]. The development of PGT techniques dates back to the late 1980s when the first successful pregnancies following IVF were reported. Initially, PGT involved the biopsy of cells from cleavage-stage embryos for genetic analysis, mainly focusing on identifying chromosomal abnormalities [2]. Over the years, technological advancements have led to the refinement of PGT techniques, including the transition from fluorescence in situ hybridization (FISH) to more sophisticated methods such as array comparative genomic hybridization (aCGH), single-nucleotide polymorphism (SNP) microarray analysis, and next-generation sequencing (NGS). These advancements have significantly improved the accuracy and reliability of PGT, facilitating better embryo selection and improving IVF success rates [1].

In modern reproductive medicine, PGT plays a crucial role in assisting couples with genetic disorders or those at risk of transmitting hereditary conditions to their offspring. PGT enables the identification and elimination of embryos carrying genetic abnormalities, thereby reducing the likelihood of genetic diseases in offspring and alleviating the emotional and financial burden of managing such conditions [3]. Moreover, PGT has expanded its applications beyond genetic disease screening to include aneuploidy screening, mitochondrial DNA testing, and embryo sex selection, further enhancing its relevance in contemporary IVF practice [4].

This comprehensive review aims to provide an in-depth analysis of the advancements and applications of PGT in IVF. By examining the historical background, evolution of PGT techniques, clinical applications, efficacy, challenges, and future directions in PGT, this review offers valuable insights into the current state of PGT in modern reproductive medicine. Additionally, the review aims to highlight the significance of PGT in improving IVF outcomes, preventing genetic diseases, and shaping the future of assisted reproductive technology.

## Review

Types of preimplantation genetic testing

PGT-A (Aneuploidy Screening)

Preimplantation genetic testing for aneuploidies (PGT-A) stands as a pivotal technique within the realm of IVF, aimed at scrutinizing embryos for specific chromosome abnormalities to enhance live birth rates through the selection of embryos bearing the correct number of chromosomes for subsequent transfer into the uterus [2,5,6]. PGT-A entails the meticulous examination of embryos conceived in vitro for aneuploidies, thereby ensuring that only euploid embryos - those exhibiting a normal chromosome count - are chosen for transfer, thereby mitigating the risks associated with miscarriage, unsuccessful transfers, or the birth of offspring with chromosomal anomalies like Down syndrome or Turner syndrome [5,6]. This screening procedure meticulously evaluates the chromosomal makeup of the embryo to detect any instances of missing or extra chromosome material, which could potentially lead to implantation failures, miscarriages, or health complications in the offspring [7]. Typically, PGT-A is recommended for patients grappling with recurrent pregnancy losses or advanced maternal age, as the latter represents a significant contributing factor to chromosomal abnormalities in embryos [6,7]. Despite the potential benefits of PGT-A in bolstering the likelihood of a successful pregnancy by identifying and selecting healthier embryos, it is imperative to acknowledge the inherent risks associated with the procedure, including the potential for misdiagnosis, the risk of embryo damage during biopsy, and the possibility of encountering a lack of viable embryos for transfer if all are found to possess abnormal chromosomes [5-7].

PGT-M (Monogenic/Single-Gene Disorder Screening)

Preimplantation genetic testing for monogenic disorders (PGT-M) represents a specialized application of preimplantation genetic diagnosis within ART and IVF, aimed at assessing embryos for specific genetic conditions to prevent the inheritance of severe disorders in the majority of cases [8,9]. PGT-M involves a biopsy conducted at the blastocyst stage, enabling the removal of five to 10 cells for genetic testing to identify unaffected embryos suitable for transfer [8]. This form of testing proves particularly advantageous for severe childhood-onset conditions lacking specific treatment or intervention, such as Tay Sachs disease, as well as for identifying significant adult-onset conditions like hereditary breast and ovarian cancer (HBOC) [8]. Furthermore, PGT-M facilitates human leukocyte antigen (HLA) matching to identify compatible embryos for potential HLA matches and to avert the transfer of affected embryos [8]. Notably, genetic counselling plays a pivotal role both before and after PGT-M, guiding decision-making regarding embryo transfer and providing prenatal diagnosis for the confirmation of testing results [8]. In essence, PGT-M is a vital tool in identifying and selecting healthy embryos for transfer, effectively reducing the risk of transmitting monogenic disorders to offspring.

PGT-SR (Structural Rearrangement Screening)

**Figure 1:**
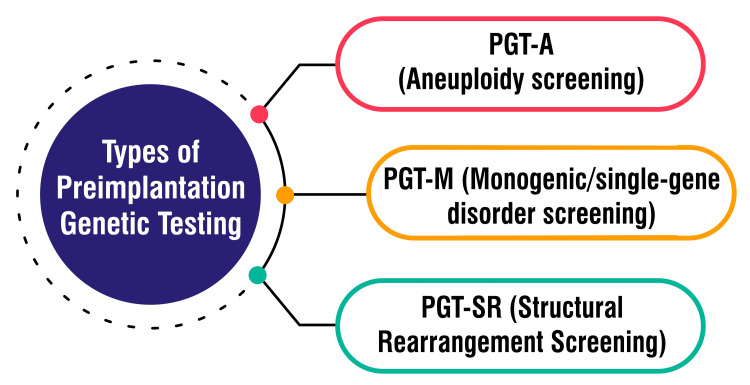
Types of preimplantation genetic testing PGT-A: Preimplantation Genetic Testing for Aneuploidies, PGT-M: Preimplantation Genetic Testing for Monogenic Disorders, PGT-SR: Preimplantation Genetic Testing for Structural Rearrangements The corresponding author Seema Yelne created this figure

PGT-SR, or preimplantation genetic testing for structural rearrangements, plays a pivotal role in genetic screening performed on embryos conceived through IVF, specifically targeting chromosomal structural rearrangements triggered by balanced translocations and inversions [10]. This testing protocol is significant for individuals harbouring chromosomal rearrangements such as inversions, reciprocal translocations, or Robertsonian translocations. This can give rise to embryos bearing missing or extrachromosomal segments. Such aberrations increase the likelihood of miscarriage, stillbirth, or the birth of offspring with severe health complications [11]. PGT-SR is designed to pinpoint embryos possessing the correct chromosomal material, thereby enhancing the prospects of establishing a healthy pregnancy and achieving a successful live birth [11]. The procedure entails a thorough analysis of embryo chromosome structure, with only those deemed normal being selected for transfer, thus elevating the overall success rate of IVF procedures for individuals predisposed to producing embryos afflicted by chromosomal abnormalities stemming from structural rearrangements [11]. Figure 1 shows types of preimplantation genetic testing.

Emerging Techniques and Future Directions in PGT

Ongoing research explores innovative approaches to enhance the accuracy and efficiency of PGT. These endeavours encompass advancements in biopsy techniques, the timing of biopsies, and genetic analysis methods, all aimed at bolstering the reliability of results [4]. NGS technology is revolutionizing PGT by furnishing more comprehensive insights into an embryo's genetic composition. By offering detailed information about chromosomal abnormalities and genetic conditions, NGS facilitates a more thorough assessment of embryos, thus augmenting the precision of PGT outcomes [12].

Karyomapping emerges as a significant technique within the realm of PGT, allowing for the simultaneous detection of aneuploidy and monogenic disorders in embryos. This approach affords a comprehensive analysis, addressing both chromosomal abnormalities and specific genetic conditions, thereby furnishing valuable insights for selecting embryos [4]. The future trajectory of PGT involves personalized testing strategies tailored to individual patients' genetic profiles and family histories. This personalized approach aims to optimize the selection of healthy embryos while mitigating the risk of transmitting genetic diseases to offspring [7]. As advancements in PGT techniques persist, there is a concerted effort to improve success rates in IVF cycles. By meticulously selecting the healthiest embryos for transfer, ongoing innovations aim to enhance the likelihood of successful pregnancies and mitigate pregnancy complications [13,14].

Advancements in PGT technologies

Next-Generation Sequencing Applications in PGT

NGS has fundamentally transformed PGT by introducing advanced capabilities in genetic analysis. NGS enables the quantitative analysis of chromosome numbers in embryos, distinguishing between euploid embryos possessing the standard 46 chromosomes [15]. This technology has markedly improved PGT by facilitating the screening of multiple samples per analysis, thereby reducing costs and opening up new diagnostic avenues, such as detecting whole chromosome aneuploidies and mosaicism [16]. NGS-based PGT has proven highly effective in screening for monogenic diseases, matching human leukocyte antigens, and detecting mosaicism more efficiently than alternative methods like SNP arrays [17,18]. Overall, the utilization of NGS in PGT has showcased superior accuracy, efficiency, and diagnostic capabilities, positioning it as a cornerstone technology in ensuring healthy pregnancies and successful births for couples undergoing IVF procedures.

Comparative Genomic Hybridization and Its Variations

Comparative genomic hybridization (CGH) is a molecular cytogenetic method employed for analyzing copy number variations (CNVs) in DNA sans the requirement for cell culturing. This technique entails comparing two genomic DNA samples to pinpoint disparities in gains or losses of chromosomal regions. Over time, CGH has evolved into more specialized forms like array CGH (aCGH), which facilitates a locus-by-locus assessment of CNVs with heightened resolution, reaching levels as low as 100 kilobases [19]. The advent of array CGH technology has proven pivotal in identifying segmental DNA copy number alterations, enabling meticulous examination for the detection of genetic modifications and copy number variations across a spectrum of applications encompassing cancer genetics, constitutional diseases, and human variation [20]. This progression has significantly bolstered the capability to comprehensively profile segmental copy number imbalances at an unparalleled resolution, positioning it as an invaluable diagnostic and investigative instrument in clinical settings [19,20]. Furthermore, CGH techniques have found utility in interspecies comparisons and have been harnessed as diagnostic aids for cancer, congenital anomalies, developmental delay, mental retardation, and the identification of chromosomal aberrations in embryos [19,20]. The ongoing refinement and application of CGH and its iterations underscore their indispensable role in genetic analysis, disease diagnosis, and research pursuits.

Single-Nucleotide Polymorphism Microarray Analysis

SNP microarray analysis emerges as a potent tool wielded across various domains, including cancer research and clinical diagnostics, to discern genetic variations at the single nucleotide level. This cutting-edge technology facilitates the detection of CNVs, loss of heterozygosity (LOH), and copy number alterations with remarkable sensitivity and resolution [21,22]. In the realm of cancer research, SNP microarrays assume a pivotal role in pinpointing genetic aberrations such as loss of heterozygosity, copy number alterations, and DNA methylation changes within cancerous cells, thereby aiding in the identification of cancer predisposition genes, oncogenes, and tumour suppressor genes [21,23]. Furthermore, SNP-based microarray analysis has demonstrated efficacy in diagnosing conditions like hydatidiform moles, showcasing its utility in detecting genetic abnormalities within clinical settings [21]. SNP microarray analysis is an invaluable genomic tool, furnishing intricate insights into genetic variations that prove instrumental in disease diagnosis, risk assessment, and treatment selection.

Fluorescence in Situ Hybridization and Multiplex PCR

FISH and multiplex PCR represent sophisticated molecular techniques in genetic analysis and diagnostics. FISH, a cytogenetic method, employs fluorescent probes to pinpoint specific DNA or RNA sequences within cells, tissues, or organisms. This technique enables the visualization of gene expression patterns, identification of chromosomal anomalies, and detection of RNA targets such as mRNA, lncRNA, and miRNA [24]. FISH plays a pivotal role across various domains, including genetic counselling, clinical medicine, species identification, and diverse research fields like cancer diagnosis, neuroscience, and gene expression analysis [24]. Conversely, multiplex PCR, a molecular biology technique, facilitates the concurrent amplification of multiple DNA targets in a single reaction. It is a robust tool for detecting various pathogens or genetic markers linked to diseases like Crohn's disease (CD) through amplifying specific DNA sequences unique to these pathogens [25]. The development of multiplex PCR protocols, coupled with multi-colour FISH (m-FISH), permits the simultaneous detection of multiple genes from major pathogens associated with CD, presenting a valuable avenue for investigating the multi-pathogenic nature of diseases such as CD and ulcerative colitis [25].

Clinical applications of PGT in IVF

Screening for Chromosomal Abnormalities

**Figure 2:**
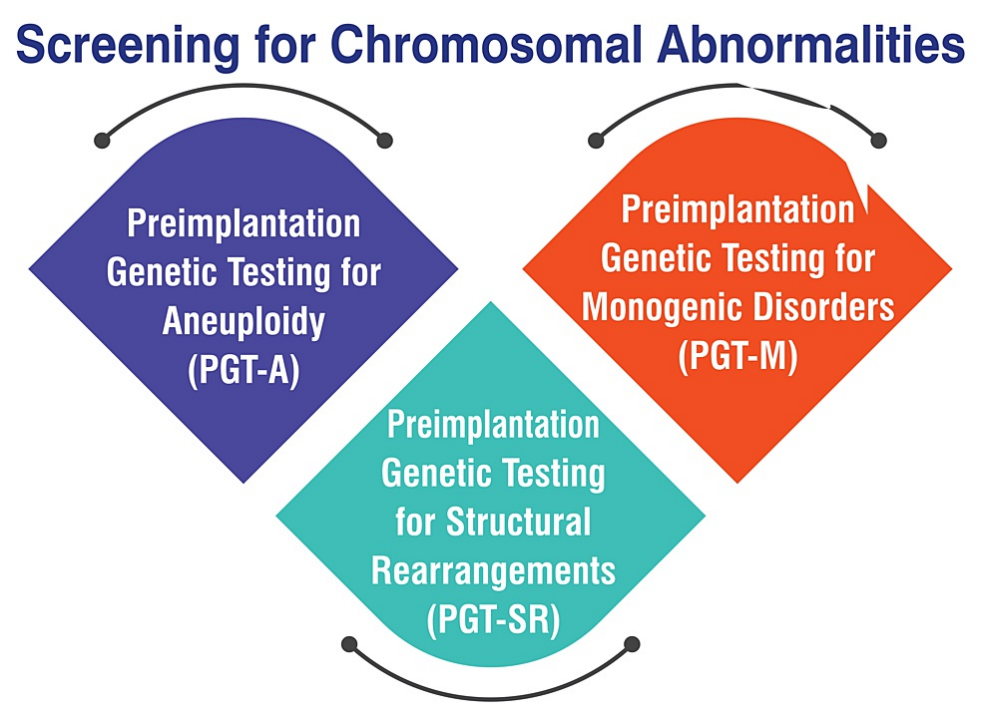
Shows screening for chromosomal abnormalities PGT-A: Preimplantation Genetic Testing for Aneuploidies, PGT-M: Preimplantation Genetic Testing for Monogenic Disorders, PGT-SR: Preimplantation Genetic Testing for Structural Rearrangements The corresponding author Seema Yelne created this figure

PGT-A is a screening process designed to detect certain chromosome abnormalities in embryos, including missing or additional chromosome material. These abnormalities can predispose embryos to risks such as miscarriage, failed transfers, or health complications in offspring [6]. PGT-M is conducted in situations where there is an elevated risk of a specific genetic condition manifesting in embryos. This testing is particularly suitable for individuals affected by a genetic disorder that could potentially be inherited by their offspring or for carriers of certain genetic conditions [6]. PGT-SR is employed when a patient or their partner exhibits chromosomal rearrangements such as translocations or inversions. These rearrangements can give rise to embryos with missing or additional chromosomal segments, thereby increasing the likelihood of pregnancy complications or serious health issues in children [6]. Figure 2 shows screening for chromosomal abnormalities.

Genetic Disease Prevention and Family Balancing

PGT offers a method to screen embryos for genetic abnormalities before implantation, thereby mitigating the risk of passing on inherited genetic disorders to offspring [26,27]. PGT-M (is specifically tailored to identify and select embryos free from specific genetic diseases such as cystic fibrosis, Tay-Sachs disease, and muscular dystrophy [27]. This form of genetic testing through PGT aids in the identification of single gene mutations associated with diseases like cystic fibrosis, spinal muscular atrophy, fragile X syndrome, sickle cell disease, and thalassemia, enabling the selection of healthy embryos for transfer [26]. PGT-A plays a critical role in screening embryos for chromosomal abnormalities, particularly in older women or individuals with a history of multiple miscarriages, thereby reducing the risk of implantation failure and miscarriage [27]. Family balancing, also referred to as gender selection, permits couples to opt for the sex of the embryo to be transferred into the uterus, allowing them to achieve a desired gender composition within their family [28,29]. PGT-A can unveil the gender of each embryo through chromosomal information, empowering couples to make informed decisions regarding the sex of their prospective children and to plan their families accordingly [28]. Family balancing through IVF is a lawful option in the United States. It holds appeal for couples seeking to have children of both genders or a specific gender for various personal reasons [28].

Mitochondrial DNA Testing

Mitochondrial DNA (mtDNA) testing is valuable across diverse fields, encompassing genealogy, forensic science, and human identification. This type of testing focuses on scrutinizing the DNA housed within mitochondria, which is solely inherited from the mother. Through mitochondrial DNA testing, individuals can gain insights into their matrilineal ancestry, facilitating the tracing of maternal lineage across generations. Particularly in genealogy research, this testing proves invaluable in identifying common ancestors and establishing connections among relatives along maternal lines [30]. In forensic science, mitochondrial DNA testing is critical in human identification. With its heightened sensitivity, mtDNA analysis can extract information from aged or degraded biological samples, proving especially vital in scenarios where nuclear DNA analysis is unfeasible. Forensic experts leverage mtDNA testing to compare samples obtained from crime scenes with those of maternally related individuals, such as siblings, thereby establishing familial relationships or verifying identities. Although mtDNA analysis cannot provide distinct identifications, it is a potent tool for acquiring information when nuclear DNA remains inaccessible [31].

Psychological and Ethical Considerations in PGT Utilization

Couples contemplating PGT encounter many factors influencing their decisions, including the aspiration for a healthy child devoid of genetic variations, financial considerations, emotional dynamics, and confidence in available technologies. The decision-making process involves cognitive evaluations, emotional reactions, and moral concerns, thereby adding layers of complexity to an already challenging scenario [32]. Ethical deliberations surrounding PGT give rise to debates encompassing issues such as eugenics, social sex (family balancing), and the selection of embryos based on gender. While some individuals voice objections to the potential misuse of PGT for prioritizing certain traits over others, others express apprehensions regarding the disposal of unused embryos and the consequences of not utilizing all embryos conceived through IVF [33].

**Figure 3:**
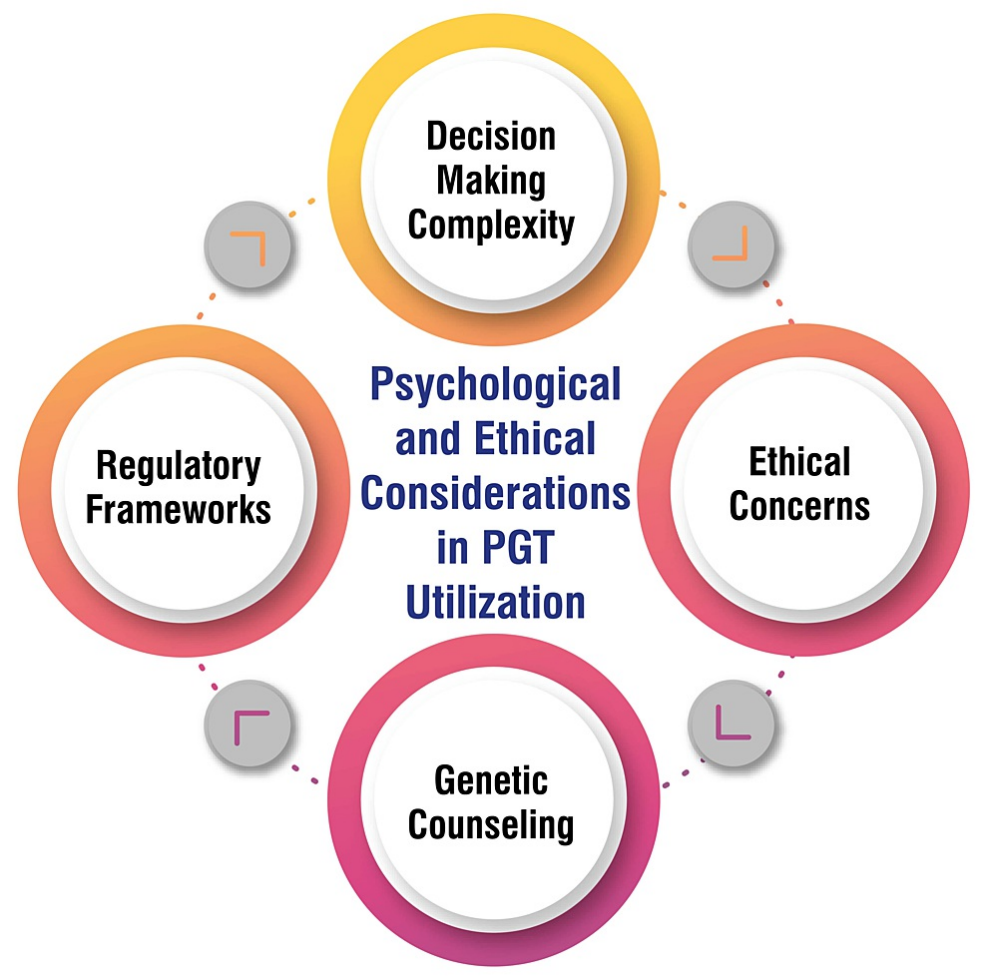
Psychological and ethical considerations in preimplantation genetic testing (PGT) utilization The corresponding author Seema Yelne created this figure

The regulatory framework governing PGT exhibits variability across nations, reflecting diverse societal perspectives and values concerning the technology. In countries like Brazil, ongoing discussions revolve around regulating preimplantation genetic diagnosis (PGD) and the necessity for new standards to ensure equitable access to this technology while addressing ethical apprehensions associated with embryo selection and genetic testing [34]. Genetic counselling assumes a pivotal role in the PGT process, aiding patients in comprehending the advantages and limitations of genetic testing, guiding them through the decision-making journey, and assisting in assessing the risks and benefits linked with PGT. It is recommended that individuals undergo genetic counselling before opting for PGT to make well-informed decisions [29]. Figure 3 shows psychological and ethical considerations in PGT utilization.

Efficacy and outcomes of PGT in IVF

Success Rates of PGT-Assisted IVF Cycles

A randomized controlled trial involving 1,212 patients revealed that PGT-A did not confer benefits to women aged 20 to 37 with a favourable prognosis for live birth compared to conventional IVF. This study raised concerns that some patients may have undergone an expensive treatment that failed to enhance their chances of achieving pregnancy through IVF [35]. A retrospective analysis of CDC data spanning from 2011 to 2012 indicated that PGD cycles aimed at aneuploidy screening resulted in a reduced risk of miscarriage for women over 35 years old and led to increased clinical pregnancy rates and live birth delivery rates among women aged over 37 years. However, no enhancement in success rates was observed for women under 35 [36]. At the Pacific Fertility Center, the implementation of PGT-A has yielded higher pregnancy rates than previous experiences. The centre advocates for PGT-A for all women undergoing IVF to optimize their chances of success. PGT-A aids in improving IVF pregnancy rates per transfer, advocating for elective single embryo transfer (eSET) to mitigate risks associated with multiple gestations and diminishing the likelihood of miscarriage and chromosomal abnormalities [37]. In a multicenter, randomized, controlled trial conducted in China, PGT-A was compared with conventional IVF in subfertile women possessing good-quality blastocysts. The study revealed that traditional IVF yielded a cumulative live birth rate non-inferior to PGT-A. Live births occurred in 77.2% of women in the PGT-A group and 81.8% in the traditional IVF group [38].

Reduction of Miscarriage Rates With PGT

PGT-A has shown promise in significantly reducing miscarriage rates, especially among women of advanced maternal age. Through PGT-A, embryos with chromosomal abnormalities can be identified and screened out, leading to a notable decrease in the risk of miscarriage. For example, in a retrospective study involving women under 35 years old, the miscarriage rate decreased from 9% in the control group to 2.6% with PGT-A [39,40]. In studies focusing on women under 38 years old with a history of one prior miscarriage and embryonic chromosomal abnormalities detected in previous products of conception, PGT-A demonstrated effectiveness in enhancing the clinical pregnancy rate per embryo transfer. Although there was no significant difference in live birth rates between the PGT-A and control groups, the higher clinical pregnancy rate suggests that PGT-A may improve the chances of achieving a successful pregnancy in this demographic [39]. Moreover, euploid embryos identified through PGT-A have been associated with lower miscarriage rates and higher success rates in IVF procedures. Comparisons between euploid and untested embryo transfers have revealed comparable miscarriage rates, emphasizing the importance of selecting chromosomally healthy embryos to mitigate the risk of miscarriage [41].

Impact of PGT on Live Birth Rates and Pregnancy Outcomes

The impact of PGT on live birth rates and pregnancy outcomes is substantial, as evidenced by research findings. PGT has been associated with improved clinical pregnancy rates and live birth rates, particularly among women over the age of 35 undergoing IVF procedures. Studies have demonstrated a statistically significant increase in the clinical pregnancy rate when PGT-A is utilized with a single blastocyst, showcasing its effectiveness in enhancing successful pregnancies [42]. Furthermore, the adoption of PGT has yielded positive outcomes, with high live birth rates observed in cycles incorporating NGS for PGT-A. Research indicates that the live birth rate per transfer following frozen single euploid embryo transfer cycles employing NGS-based PGT-A remained consistently high across various maternal age groups, underscoring the reliability and effectiveness of PGT in improving pregnancy outcomes [42]. PGT plays a pivotal role in augmenting the success rates of IVF procedures by selecting healthy embryos for transfer, mitigating the risk of genetic diseases in offspring, and heightening the likelihood of a successful pregnancy. The findings from studies highlight the favourable impact of PGT on live birth rates and pregnancy outcomes, solidifying its position as a valuable tool in assisted reproductive technologies for ensuring healthier pregnancies and births.

Long-Term Follow-Up Studies on PGT-Conceived Children

Long-term follow-up studies on children conceived through PGT have provided invaluable insights into their health outcomes. These studies, conducted up to the ages of four to six years, have yielded reassuring results concerning the cognitive development and overall health of PGT-conceived children. Research indicates that children born after PGT exhibit perinatal outcomes comparable to those born after traditional IVF procedures, highlighting the safety and efficacy of PGT in assisted reproduction [43,44]. Furthermore, these studies underscore the importance of conducting additional long-term follow-up research on children born after PGT, considering the invasiveness of the technique and the necessity to evaluate any potential risks or benefits associated with PGT. While specific findings suggest a potential association between embryo biopsy for PGT and specific health outcomes such as low birth weight and preterm birth, the overall evidence does not definitively support an increase in adverse obstetric, neonatal, or long-term outcomes in children conceived through PGT [43,45].

Challenges and limitations of PGT

Technical Challenges and Limitations of Current PGT Methods

**Figure 4:**
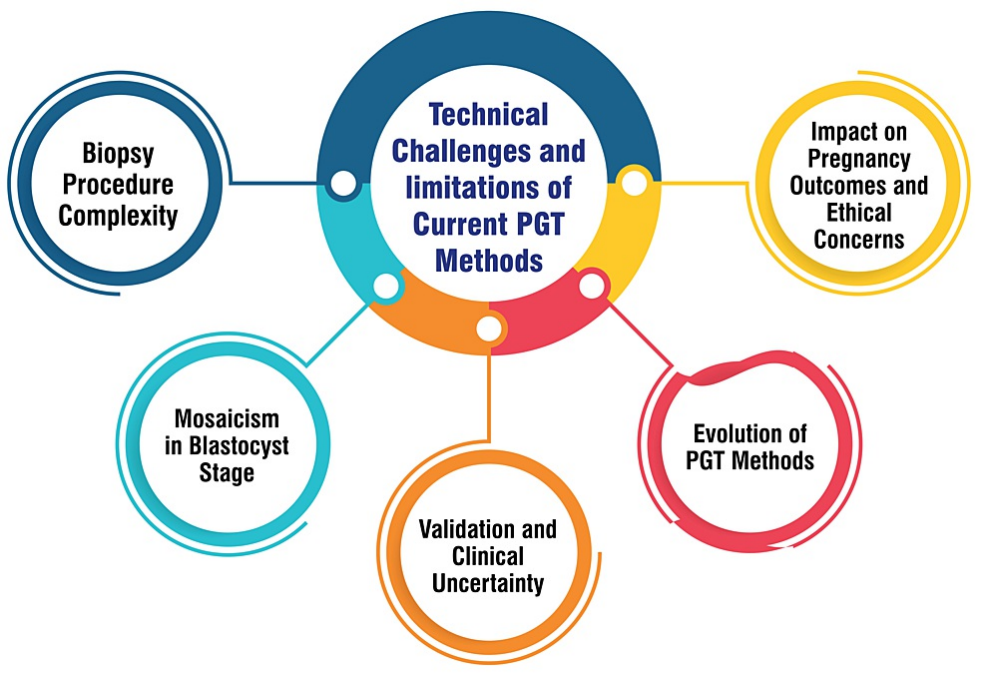
Technical challenges and limitations of current preimplantation genetic testing (PGT) methods The corresponding author Seema Yelne created this figure

PGT involves a biopsy from the embryo, necessitating precision to ensure minimal harm to the embryo. This procedure's complexity lies in its requirement for meticulous execution to safeguard the embryo's safety [46]. Mosaicism, characterized by a mix of normal and abnormal cells within an embryo, is more prevalent in the blastocyst stage during PGT-A than previously acknowledged. This heightened occurrence challenges accurately identifying embryos with genetic abnormalities [46]. PGT-A functions as a screening test rather than a diagnostic one and lacked clinical validation before its widespread utilization in patients. Uncertainty persists regarding the optimal application of this technology across specific age groups, underscoring existing knowledge gaps that necessitate addressing for more effective implementation [47]. The evolution of PGT methods, progressing from FISH to advanced techniques such as aCGH, SNP arrays, and NGS, has introduced new challenges concerning accuracy, result interpretation, and clinical validation [48]. Beyond technical hurdles, ongoing debates revolve around the impact of PGT on pregnancy outcomes and ethical considerations associated with selecting embryos based on genetic testing methods. These discussions require careful deliberation to address practical and ethical concerns [49]. Figure 4 shows the technical challenges and limitations of current PGT methods.

Ethical Concerns Surrounding Embryo Selection and Genetic Manipulation

In the realm of genetic manipulation, one ethical consideration centres on the challenge of obtaining informed consent from embryos, which cannot provide consent for genetic interventions. This dilemma raises pertinent questions regarding autonomy and decision-making in the context of genome editing [50]. Discussions surrounding the moral status of human embryos are pivotal in shaping ethical perspectives on genetic manipulation. While some argue for the intrinsic moral value of embryos, others emphasize the potential benefits of genome editing in preventing genetic diseases [50].

**Figure 5:**
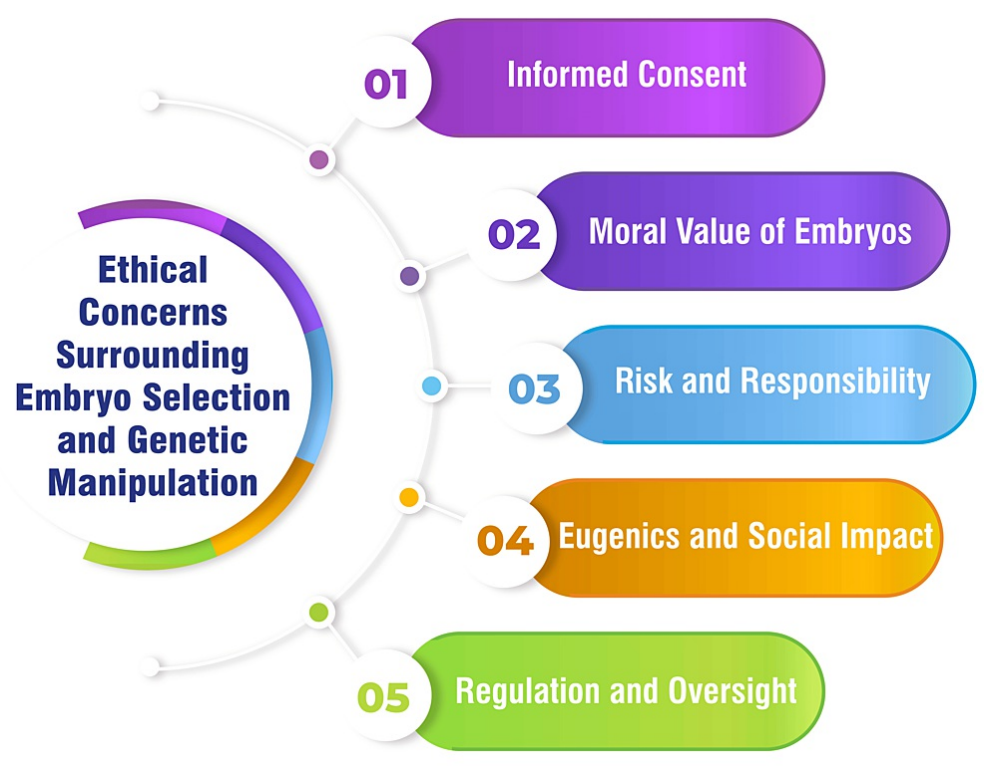
Ethical concerns surrounding embryo selection and genetic manipulation The corresponding author Seema Yelne created this figure

Despite advancements like CRISPR/Cas9 reducing the risks associated with genetic modification technologies, thorough evaluation of potential consequences and managing uncertainties are imperative before clinical applications. Assessing risk and responsibility remains a crucial aspect of navigating ethical considerations [50]. Concerns about eugenics and the broader societal implications of genetic manipulation prompt reflections on inequality, stigma, and the impact on individuals with genetic disabilities. The availability and affordability of genetic modifications can exacerbate existing social disparities and pose new challenges in healthcare access and societal norms [51,52]. Ensuring robust regulatory frameworks to govern genetic technologies, including PGD, is essential to address ethical dilemmas and ensure responsible and equitable application. Societies grapple with the delicate balance between individual freedoms and the social consequences of genetic manipulation [51]. Figure 5 shows ethical concerns surrounding embryo selection and genetic manipulation.

Cost-Effectiveness and Accessibility Issues

A systematic review scrutinizing the cost-effectiveness analyses of PGT underscores the importance of evaluating these studies based on specific criteria to gauge their overall quality. This comprehensive review likely offers valuable insights into the economic implications of PGT within the context of IVF treatments [53]. Evidence suggests that PGT for aneuploidy proves cost-effective, reduces treatment duration, and diminishes the likelihood of failed embryo transfer. Such findings indicate that investing in PGT could yield improved outcomes while potentially curtailing the overall costs associated with IVF treatments [54,55]. Moreover, cost-effectiveness analyses have delved into the economic feasibility of PGT-M for addressing specific gene mutations such as BRCA1 and BRCA2. These analyses posit that IVF coupled with PGT-M may be economically viable for mutation carriers, reinforcing the financial advantages of integrating genetic testing into IVF procedures [56]. Additionally, incorporating preimplantation genetic screening (PGS) into IVF has demonstrated the potential to bolster live birth rates in older women. This underscores the potential cost-effectiveness of leveraging genetic screening technologies to enhance the success rates of IVF procedures [57].

Future Prospects for Overcoming Challenges in PGT Implementation

Future advancements in PGT technology promise improved accuracy, particularly with the advancement of NGS techniques. These developments are anticipated to enhance the identification of genetic abnormalities in embryos, thereby reducing the risk of false results and bolstering the overall reliability of PGT [4]. As PGT continues to expand, potentially encompassing the selection of desirable traits in embryos, establishing clear ethical guidelines and regulations will be imperative. Ethical considerations about eugenics and the selection of specific genetic traits must be addressed to ensure the responsible and ethical utilization of PGT [49].

**Figure 6:**
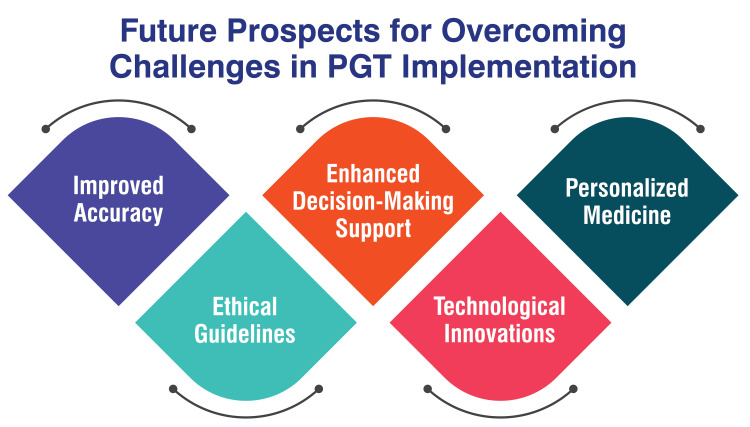
Future prospects for overcoming challenges in preimplantation genetic testing (PGT) implementation The corresponding author Seema Yelne created this figure

Ensuring comprehensive genetic counselling and support for patients undergoing PGT will be vital. Prospects may entail integrating advanced counselling services to assist individuals in navigating the intricate decision-making process associated with PGT, considering its advantages and limitations [29]. Technological innovations in biopsy techniques, biopsy timing, and data analysis methods are poised to refine the accuracy and efficiency of PGT. Progress in these areas has the potential to streamline the PGT process, rendering it more accessible and dependable for patients undergoing IVF [29]. The future landscape of PGT may shift towards personalized medicine, wherein genetic testing is tailored to individual patient's needs and genetic profiles. This customised approach could optimize the selection of healthy embryos for transfer, thereby augmenting the success rates of IVF procedures [29]. Figure 6 shows future prospects for overcoming challenges in PGT implementation.

Future directions and innovations in PGT

Advancements in Non-invasive PGT Techniques

Advancements in non-invasive PGT techniques have profoundly reshaped the landscape of assisted reproductive technologies, aiming to bolster the safety, precision, and efficiency of genetic testing within the realm of IVF. One pivotal advancement lies in developing non-invasive approaches that scrutinize cell-free DNA (cfDNA) extracted from the blastocoel or embryo culture media, thus preventing the need for invasive embryo biopsies. This innovative method facilitates the evaluation of genetic status without directly impacting the embryo, mitigating the risk of damage and enhancing the overall viability of embryos [58,59]. Moreover, integrating sophisticated technologies such as NGS and SNP arrays into non-invasive PGT techniques has facilitated the simultaneous assessment of monogenic and chromosomal abnormalities at heightened resolution. These cutting-edge technologies furnish a more comprehensive analysis of genetic information, encompassing mosaicism, structural aberrations, segmental abnormalities, and chromosomal deletions, thereby augmenting the precision and depth of genetic testing outcomes [58,60]. In essence, the evolution of non-invasive PGT techniques signifies a significant leap forward in the domain of assisted reproduction, furnishing a safer and more patient-centric approach to genetic testing during IVF cycles. These advancements elevate the accuracy and reliability of genetic analysis and mitigate the emotional stress and physical risks associated with traditional invasive procedures, ultimately enriching IVF procedures' success rates and outcomes.

Integration of Artificial Intelligence and Machine Learning in PGT Analysis

Integrating artificial intelligence (AI) and machine learning into PGT analysis heralds a cutting-edge approach brimming with significant potential and notable challenges. Recent studies have delved into the utilization of AI in PGT-A, particularly in predicting aneuploidy in embryos, shedding light on crucial epistemic and ethical considerations. The application of AI in PGT entails harnessing algorithms crafted using neural networks and machine learning to sift through vast troves of data generated by time-lapse systems to enhance the accuracy of predicting euploidy and aneuploidy in embryos [61]. Nevertheless, the incorporation of AI in PGT analysis unfurls critical concerns. Chief among them is the opacity inherent in AI models, rendering them arduous to decipher and potentially paving the way for biased predictions or errors. The absence of transparency in AI algorithms gives rise to significant epistemic and ethical quandaries, encompassing information asymmetries, the peril of misrepresenting vital values, and the prospect of adverse outcomes in embryo selection and patient welfare [62]. While the potential of AI in PGT analysis holds promise, it is imperative to tread cautiously and uphold transparency. Ongoing research indicates that AI can aid in identifying priority embryos for transfer; however, the complete deployment of AI for detecting aneuploidies in embryos necessitates further fine-tuning and careful deliberation regarding the epistemic and ethical ramifications entailed. The trajectory of AI and machine learning in PGT analysis brims with potential but demands continual exploration and meticulous assessment to guarantee its safe and efficacious assimilation into assisted reproductive technologies like IVF [62].

Potential Applications of CRISPR/Cas9 Gene Editing Technology in PGT

**Figure 7:**
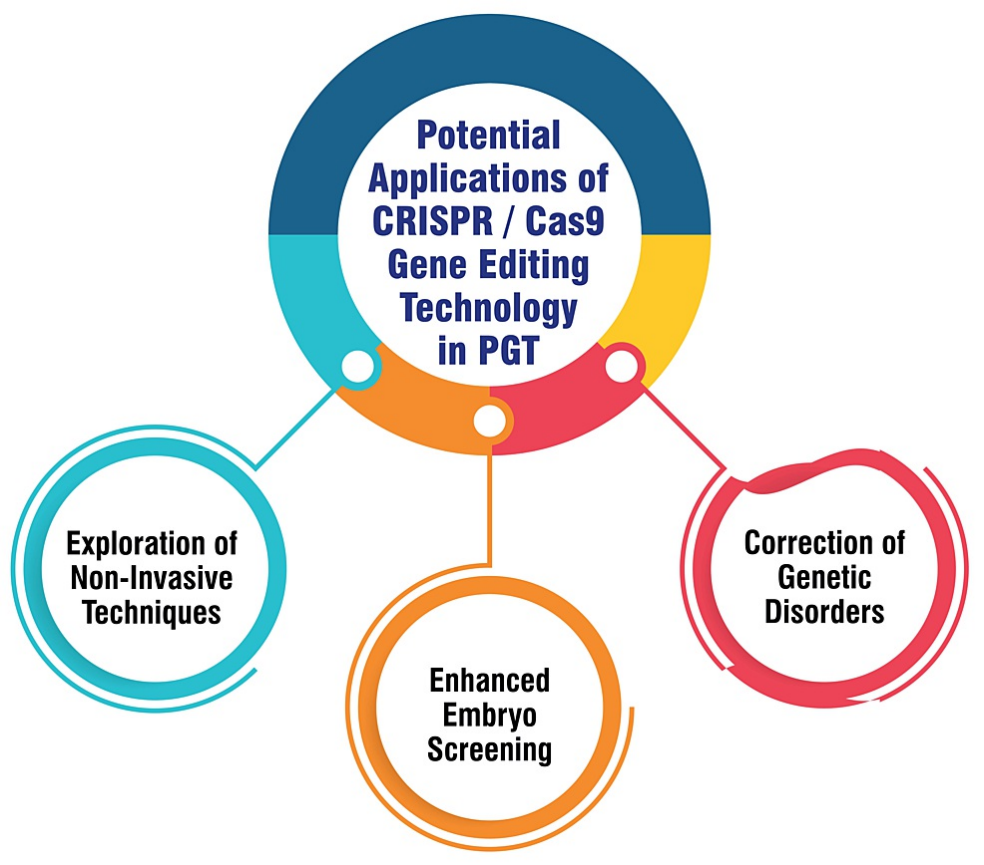
Potential applications of CRISPR/Cas9 gene editing technology in preimplantation genetic testing (PGT) The corresponding author Seema Yelne created this figure

Utilizing CRISPR/Cas9 technology holds immense potential in correcting genetic disorders within embryos, thereby mitigating the risk of hereditary diseases being passed down to subsequent generations. This innovative technology provides a precise and efficient means of editing the DNA of embryos before implantation, offering a proactive approach to preventing the transmission of genetic disorders [63,64]. Moreover, CRISPR/Cas9 enables researchers to target specific genes linked to genetic abnormalities, allowing for precise edits to ensure the selection of healthy embryos for transfer during IVF procedures. This targeted approach enhances the accuracy and effectiveness of embryo screening in PGT procedures, potentially improving pregnancy outcomes [63]. Furthermore, exploring CRISPR/Cas9 technology opens avenues for developing non-invasive techniques for genome editing in embryos. By minimizing the risks associated with invasive biopsy procedures, this advancement could revolutionize PGT by enhancing safety and efficiency in embryo screening processes. Such innovations have the potential to significantly impact assisted reproductive technologies, offering new possibilities for ensuring healthy pregnancies and births [65]. Figure 7 shows potential applications of CRISPR/Cas9 gene editing technology in PGT.

Personalized Medicine Approaches in PGT for Enhanced Embryo Selection

Personalized medicine approaches within PGT are geared toward refining embryo selection by identifying genetic abnormalities in embryos produced through IVF. PGT facilitates the identification and selection of embryos devoid of specific genetic conditions, lowering the risk of genetic diseases in prospective offspring. This state-of-the-art procedure involves extracting a small subset of cells from embryos, which undergo genetic abnormality analysis in specialized laboratories such as Natera and Cooper [64]. A pivotal component of personalized medicine in PGT involves preventing the birth of children afflicted with terminal or chronic diseases and addressing familial genetic conditions. The decision to undergo PGT is influenced by multifaceted factors, encompassing the desire to avert the inheritance of genetic disorders and the influence of religious beliefs on accepting PGT as a prenatal option. Patients commonly opt for PGT to mitigate the risk of specific genetic conditions like neurofibromatosis type 1, Marfan syndrome, cystic fibrosis, sickle cell anaemia, or fragile X syndrome [65]. Furthermore, the advancement of PGT incorporates progressions in non-invasive techniques to bolster safety and efficiency. Exploration into non-invasive PGT methods seeks to diminish the invasiveness associated with traditional biopsy procedures, potentially transforming the field by furnishing more precise and dependable genetic information for embryo selection [66].

## Conclusions

In conclusion, the review highlights significant advancements and findings in PGT technology within the context of IVF. The evolution of techniques such as NGS and CGH has notably improved the accuracy and reliability of genetic testing in embryos, broadening the utility of PGT beyond aneuploidy screening to encompass monogenic disorder detection. The implications of PGT for the future of ART are profound, with the potential to enhance IVF success rates, reduce the incidence of genetic disorders, and alleviate the emotional and financial burdens associated with failed pregnancies and genetic diseases. Looking ahead, personalized medicine approaches and tailored embryo selection strategies hold promise for further revolutionizing the field of ART. Recommendations for clinicians, researchers, and policymakers include staying updated on the latest PGT techniques and guidelines, exploring innovative technologies, establishing clear regulatory frameworks, and fostering collaboration to maximize the potential benefits of PGT in assisted reproduction. By adhering to these recommendations, stakeholders can contribute to the responsible and effective integration of PGT into clinical practice, ultimately improving outcomes for individuals and families undergoing IVF.
